# Mating Behavior of *Daphnia*: Impacts of Predation Risk, Food Quantity, and Reproductive Phase of Females

**DOI:** 10.1371/journal.pone.0104545

**Published:** 2014-08-11

**Authors:** Geung-Hwan La, Jong-Yun Choi, Kwang-Hyeon Chang, Min-Ho Jang, Gea-Jae Joo, Hyun-Woo Kim

**Affiliations:** 1 Department of Environmental Education, Sunchon National University, Suncheon, Korea; 2 Department of Life Sciences, Pusan National University, Busan, Korea; 3 Department of Environmental Sciences and Engineering, Kyung Hee University, Gyeonggi, Korea; 4 Department of Biology Education, Kongju National University, Gongju, Korea; University of Missouri, United States of America

## Abstract

High predation risk and food depletion lead to sexual reproduction in cyclically parthenogenetic *Daphnia*. Mating, the core of sexual reproduction, also occurs under these conditions. Assessment of the environmental conditions and alteration of mating efforts may aid in determining the success of sexual reproduction. Here, we evaluated the impacts of predation risk, food quantity, and reproductive phase of females on the mating behavior of *Daphnia obtusa* males including contact frequency and duration using video analysis. Mating–related behavior involved male–female contact (mating) as well as male–male contact (fighting). Mating frequency increased while unnecessary fighting decreased in the presence of predation risk. In addition, low food concentration reduced fighting between males. Males attempted to attach to sexual females more than asexual females, and fighting occurred more frequently in the presence of sexual females. Duration of mating was relatively long; however, males separated shortly after contact in terms of fighting behavior. Thus, assessment of environmental factors and primary sexing of mates were performed before actual contact, possibly mechanically, and precise sex discrimination was conducted after contact. These results suggest that mating in *Daphnia* is not a random process but rather a balance between predation risk and energetic cost that results in changes in mating and fighting strategies.

## Introduction

*Daphnia* (Cladocera) exhibit two types of reproductive modes: asexual and sexual. Asexual reproduction is performed under favorable conditions such as low predation pressure and adequate food, and through this reproductive mode, adult females produce clones (both females and males) of themselves without mating [1]. However, sexual reproduction, as an alternative to prevent the extermination of population, is required since *Daphnia* inhabit changing environments in which high seasonal mortality occurs due to predation as well as food depletion caused by rapidly increasing conspecifics and other zooplankton. Sexual reproduction is triggered in *Daphnia* populations in response to biological signals, including predation risk mediated by chemicals (kairomones) released by various predators [2], food shortage, or quality deterioration [3], [4] and crowding of conspecifics [5]. In addition to these biological factors, environmental factors such as temperature [6] and photoperiod [7] also promote sexual reproduction.

During sexual reproduction, females produce haploid sexual eggs in their ovaries instead of immediately hatching asexual eggs. In order to develop, sexual eggs must be fertilized through mating, although hatching can be postponed for many years depending on the conditions [8]. Fertilized diploid eggs are enclosed within a dark, protective shell called the ephippium before they are released into the water column. Unfertilized eggs are possibly resorbed and only the empty ephippium is produced [9]. Resting eggs in the ephippium are resistant against harsh conditions such as the digestive enzymes of fish [10] and drying, although their decreases viability over time [11]. Resting eggs hatch during favorable periods (e.g. spring) and ensure the persistence of the population [12]. In addition, they give rise to genetic diversity in the population by gene recombination [13].

As a core element of sexual reproduction, mating of cladoceran involves the processes of encounter, grasp, and copulation [14]. Among these, the encounter rate is strongly affected by the population density [15]. In general, males are scarce in parthenogenetic *Daphnia* populations since the sex ratio is biased toward females. Moreover, *Daphnia* distribute sparsely in their habitatand have poorly developed eyesight; they can only distinguish changes in light intensity [16] and detect a limited light spectrum in the turbid water [17]. Thus, both males and sexual females may encounter problems in finding each other due to their limited visual sensory abilities. *Daphnia* employ several techniques to overcome this. First, as a life history change, the production of large quantities of males during sexual reproduction maximizes efficient mating and reduces critical density for mating [18], [19]. Further, the ultimate goal of mating in *Daphnia* is the production of sexually fertilized eggs. For this reason, males occurred 1–2 weeks before sexual females to ensure encounters between receptive females and mature males [20]. Cladocerans, such as *Moina affinis* [21] and *D. magna* [22] often form swarms (high–density distributions) consisting of both sexes in their habitats. In addition, *D. pulicaria* [23] and *Bosmina longirostris* [24] exhibit sex–dependent swimming patterns; males swim faster and exhibit scanning behavior to ensure convergence of the swimming tracks of both sexes. Although the major purpose of swarming is suggested to be predation avoidance, these life history and behavioral changes may increase encounters between the sexes. As shown in another method, females may excrete sex-related pheromones similar to the copepods [25], [26] and rotifer [27] in order to increase successive mating with conspecies.

Several environmental factors can affect mating behavior by altering the encounter rate in *Daphnia*. Predator kairomones are the ultimate factor triggering predator avoidance responses as well as evoking behavioral and life history changes in prey [28]. Male and female *Daphnia* select different distribution depths in the presence of fish kairomones [23], [29]. Moreover, the presence of predator kairomones alters the swimming speed of *Daphnia* [30], and makes them sensitive to fluid disturbances, thereby enabling faster escape responses [31]. These behavioral changes may influence the encounter rate or grasping for copulation in sexually reproducing *Daphnia*. Another candidate for alteration of the encounter rate is the food concentration. *D. magna* displays fast swimming with straightened tracks when ambient food is adequate [32]. Food quantity regulates the distribution density in cladocerans such as *D. pulex* [33] and *B. longispina* [34]; they disperse when the food supply necessary for maintaining swarms is inadequate. Thus, food quantity may influence mating behavior through changes in the encounter rate. The fast swimming in males during mate searching resulted in an increase in the encounter rate with predators [35]. *Daphnia* are preferred prey, and large females [36], especially sexual females with ephippium, are vulnerable to visual predators [37]. Therefore, the rapid identification of receptive females (sexual females) among mixtures of both sexes is important to males, as well as sexual females.

Many researchers have focused on the initiation of sexual reproduction in *Daphnia* by environmental factors such as predation risk [38], food availability [39], and post–mating processes such as hatching of sexual eggs [40], [41]. To the best of our knowledge, however, the interactive effects of environmental factors on the mating behavior of *Daphnia* have not yet been evaluated. In this study, we induced male and sexual female offspring from a clone of *D. obtusa* in the laboratory. We then observed mating behaviors of *Daphnia* and examined the effects of predation risk, food quantity, and females in different reproductive phases (asexual or sexual). The main objective of this study was to investigate the mating strategy of *Daphnia* according to environmental conditions. Particularly, we hypothesized that *Daphnia* males display reduced mating-related behaviors under predation risk and low food condition, and they show an ability to distinguish receptive females in the phase of sexual reproduction.

## Materials and Methods

### Ethics Statement

Field collections of *Daphnia* and fish in protected wetlands were carried out under the permission of the Ministry of Environment, and the field studies did not involve endangered or protected species. The Institutional Animal Care and Use Committee, which supervises animal experimentation at Sunchon National University, was not established when this study was conducted. Thus, our experiment was conducted without any type of committee approval. However, we maintained our experimental fish in a 560 L glass tank under room temperature with recirculation facility, and they were fed *ad libitum* with commercial frozen krill to comply with the animal protection laws of Korea. Fish was not sacrificed or released back to the Upo Wetlands and were maintained in the laboratory after the experiment.

### Culture

*D. obtusa* is one of the most abundant species found in the Upo Wetlands in South Korea. They typically appear during spring and diminish after an extensive production of resting eggs in early summer. A clone used in mating experiments was isolated from the Upo Wetlands (35° 18' 8.58" N, 128° 41' 15.00" E), and maintained in an incubator at 20°C under a 12 h:12 h light–dark cycle. Basically, all cultures were kept in 500 mL transparent polycarbonate beakers containing Elendt M4 medium [42]. Small green algae *Chlorella vulgaris* (strain number, UMACC 001) was added to the medium to achieve 2.5 mg·CL^−1^ [43] during daily medium change.

### Induction of male and sexual female *Daphnia*

10-day old *D. obtusa* females from culture were subjected to a high-density environment (200 individuals per 500 mL of medium) for male production. Neonates of the second clutch were collected. After 4–5 days, they were separated according to sex. Small, reddish, and fast-swimming males were maintained under a low-density regime (50 individuals per 500 mL of medium) until used. One half of the females was subjected to a high-density regime with no medium changes from day 9 in order to obtain sexual females with well-developed ovaries and immature white ephippium. The other half of females, which were kept under low-density conditions, was used as asexual females. During daily medium change, too small or large individuals due to variations in molting time were excluded. We used 12-day old sibling males as well as asexual and sexual females from the same clone for the mating experiment.

### Factors for mating experiment

Fish kairomones were obtained from one large–mouth bass (*Micropterus salmoides*; total length *c*.15 cm), a species common in the Upo Wetlands. The fish was exposed to 10 L of Elendt M4 medium for 24 hours. Then, the medium was filtered through a 0.45 µm filter and stored at −20°C until used. Food concentrations were adjusted to low (0.5 mgCL^−1^) and high (2.5 mgCL^−1^) using *C. vulgaris*. Asexual or sexual females obtained from the culture were used in the experiment.

### Observation of mating behavior

**Figure 1:**
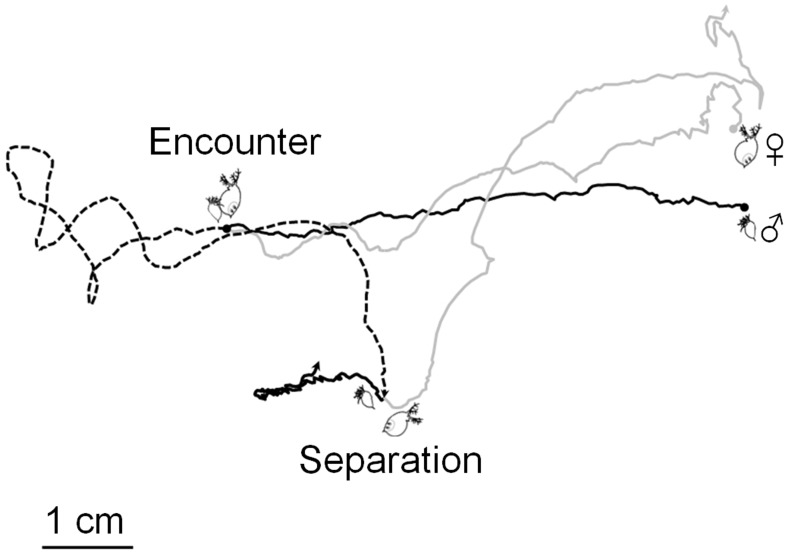
Example of video analysis of the mating process in *Daphnia obtusa*. Frame numbers of the video from encounter to separation of each contact were converted into time to determine the duration of the contact (black line: trajectory of male; grey line: trajectory of female; dashed line: trajectory of male and female during mating).

To evaluate the impact of predation risk (absence or presence of kairomones), food quantity (low or high), and reproductive phase of females (asexual or sexual) on mating, we conducted a 2×2×2 experimental design (12 replicates for each treatment, total of 96 observations). Small acryl chambers designed for two–dimensional observations were used (length × height × width  = 8.5×7×0.5 cm). The observation chamber was placed in a box made of black panel without a cover or front side. All experiments were conducted during day time and a 20 W fluorescent lamp was placed above the box for illumination. We filled the observation chamber with 30 mL of medium for each treatment (absence or presence of fish kairomones and low or high food) and added 10 males and females (asexual or sexual). Before recording, there was an acclimation period of 10 minutes. We recorded all contact events for 10 minutes under each treatment using a digital camcorder (Sanyo Xacti VPC–SH1, Tokyo, Japan) recording at 30 frames per second. Contact frequency and frame number of the video for initiation and separation of every contact event were recorded (Fig. 1). Total frame numbers of each contact were used to calculate the duration time of contact. Simple collisions (when individuals swam in a different direction immediately without adherence) and contact events occurring before the recording or continued after 10 minutes were excluded.

### Statistical methods

We analyzed contact frequency and duration with respect to fish kairomones, food quantity, and female reproductive phase using analysis of variance (ANOVA, SPSS, ver. 21).

## Results

### Mating and fighting in *D. obtusa*

**Figure 2:**
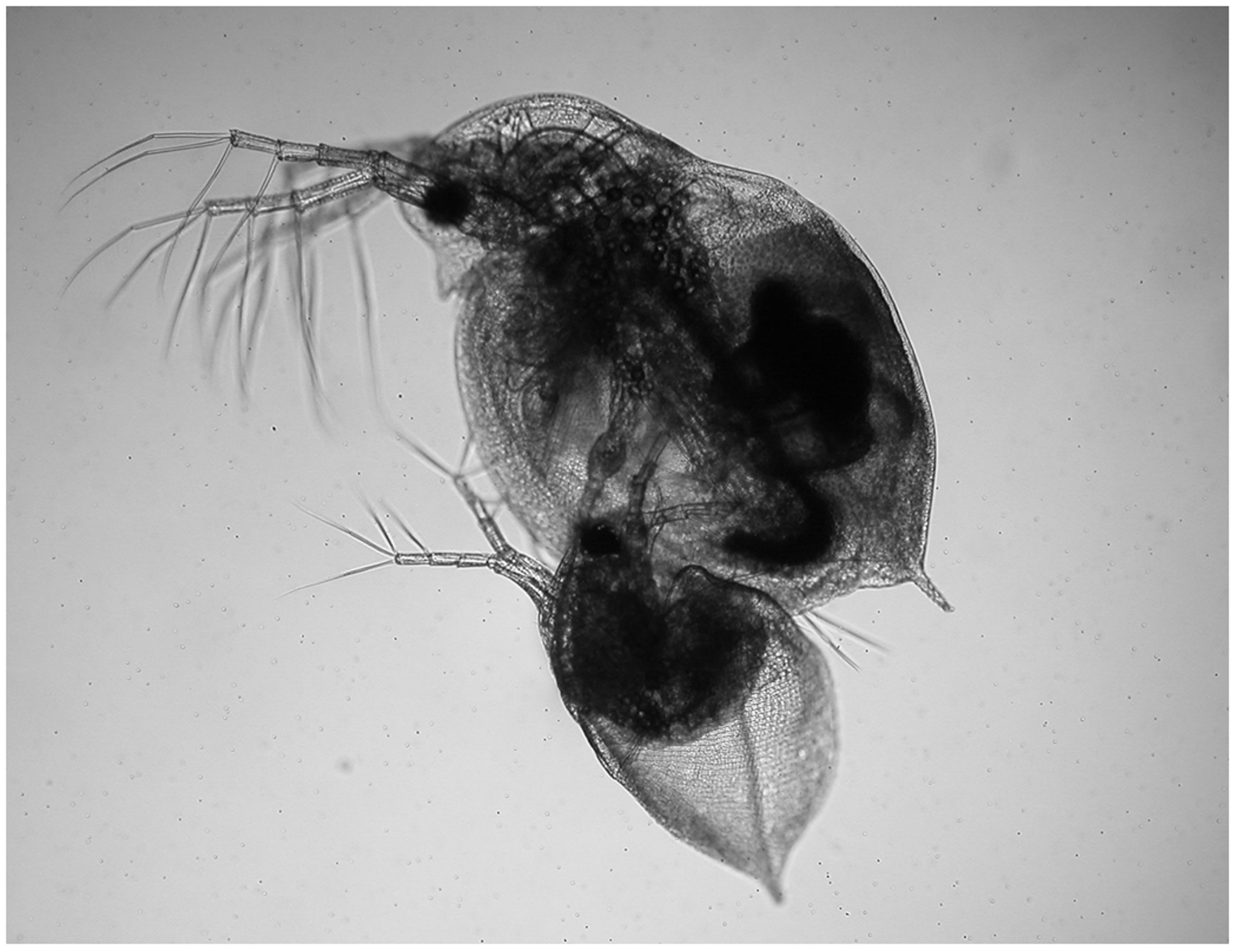
Typical mating position of *Daphnia obtusa*. Male (below) grasps the female (above) with freshly ovulated sexual eggs in the immature ephippium.

Mating behavior in *D*. *obtusa* involved two types of contact; male–female contact (mating) and male–male contact (fighting). Males frequently changed their swimming direction toward individuals that passing nearby and chased the tracks of females or other males. Normally, mating behavior involved a strong escape response with fast swimming until the male became detached from the female. This reaction was also observed in fighting. Initially, males approached females from various directions, grasped a female with its modified hook-like first antennules, positioned itself on the ventral side, and attempted to insert its abdominal claw into the carapace valve of the female for insemination (Fig. 2).

### Impact of predation risk

**Figure 3:**
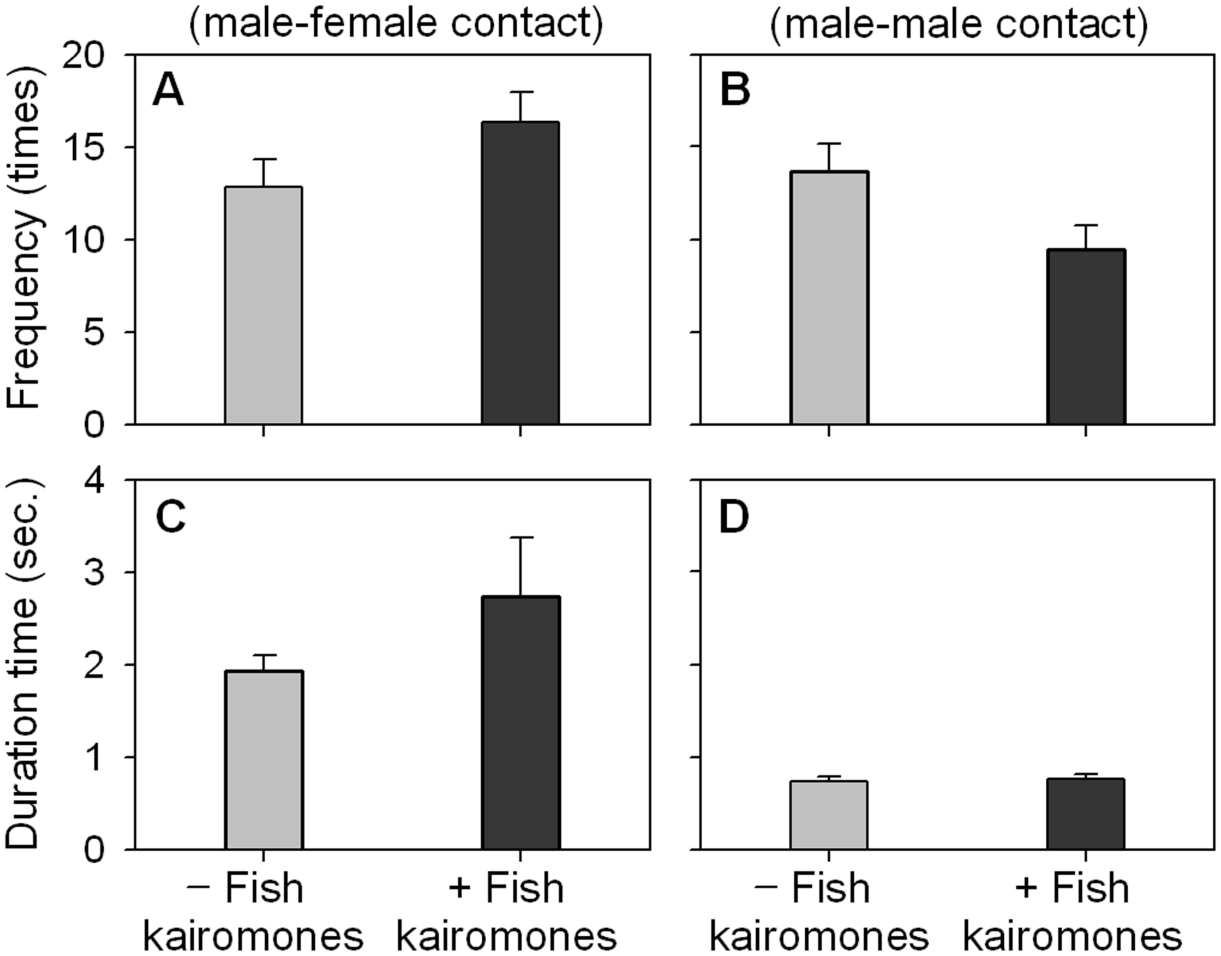
Contact frequencies and duration times of mating and fighting of *Daphnia obtusa* according to the absence or presence of fish kairomones. Observation was performed for 10 minutes. Each column represents the mean ± SE of 48 replicates.

Fish kairomones had obvious and opposite impacts on the frequencies of both mating and fighting. In mating, the mean total contact frequency significantly (*p* = 0.004) increased from 12.9 to 16.4 in the presence of fish kairomones. On the other hand, the mean total contact frequency during fighting significantly (*p* = 0.000) decreased from 13.7 to 9.5 (Fig. 3A, B and Table S1) in the presence of fish kairomones. There was no statistical difference in duration time of mating between the control and fish kairomones treatments. Similarly, there was no effect of predation risk on the duration of fights. However, fights were distinctively shorter (0.7∼0.8 sec.) than male–female contact (Fig. 3C, D and Table S1).

### Impact of food quantity

**Figure 4:**
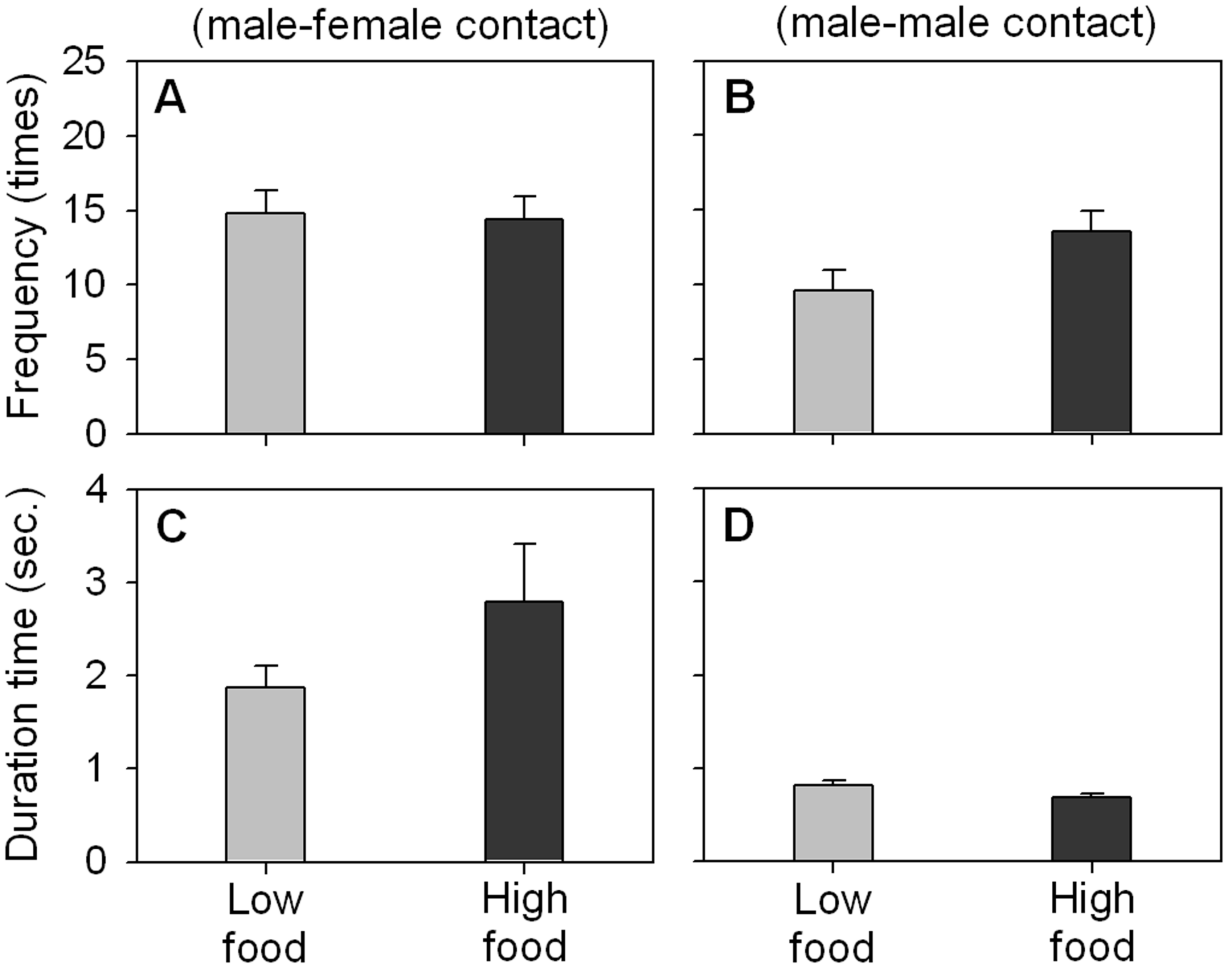
Contact frequencies and duration times of mating and fighting of *Daphnia obtusa* according to food quantity. Observation was performed for 10 minutes. Each column represents the mean ± SE of 48 replicates.

In terms of mating contacts, *D. obtusa* exhibited almost identical frequencies regardless of food quantity (14.8 at low food and 14.5 at high food) (Fig. 4A and Table S1). In contrast to the effect of fish kairomones, food quantity had a significant (*p* = 0.000) impact on the contact frequency of fighting; males tended to attach to other males more frequently when the ambient food concentration was high (9.6 under low food *vs.* 13.5 under high food conditions) (Fig. 4B and Table S1). Food quantity had no effect on the duration time. The duration of fighting was short, and both males separated quickly regardless of food quantity (Fig. 4C, D and Table S1).

### Impact of reproductive phase of females

**Figure 5:**
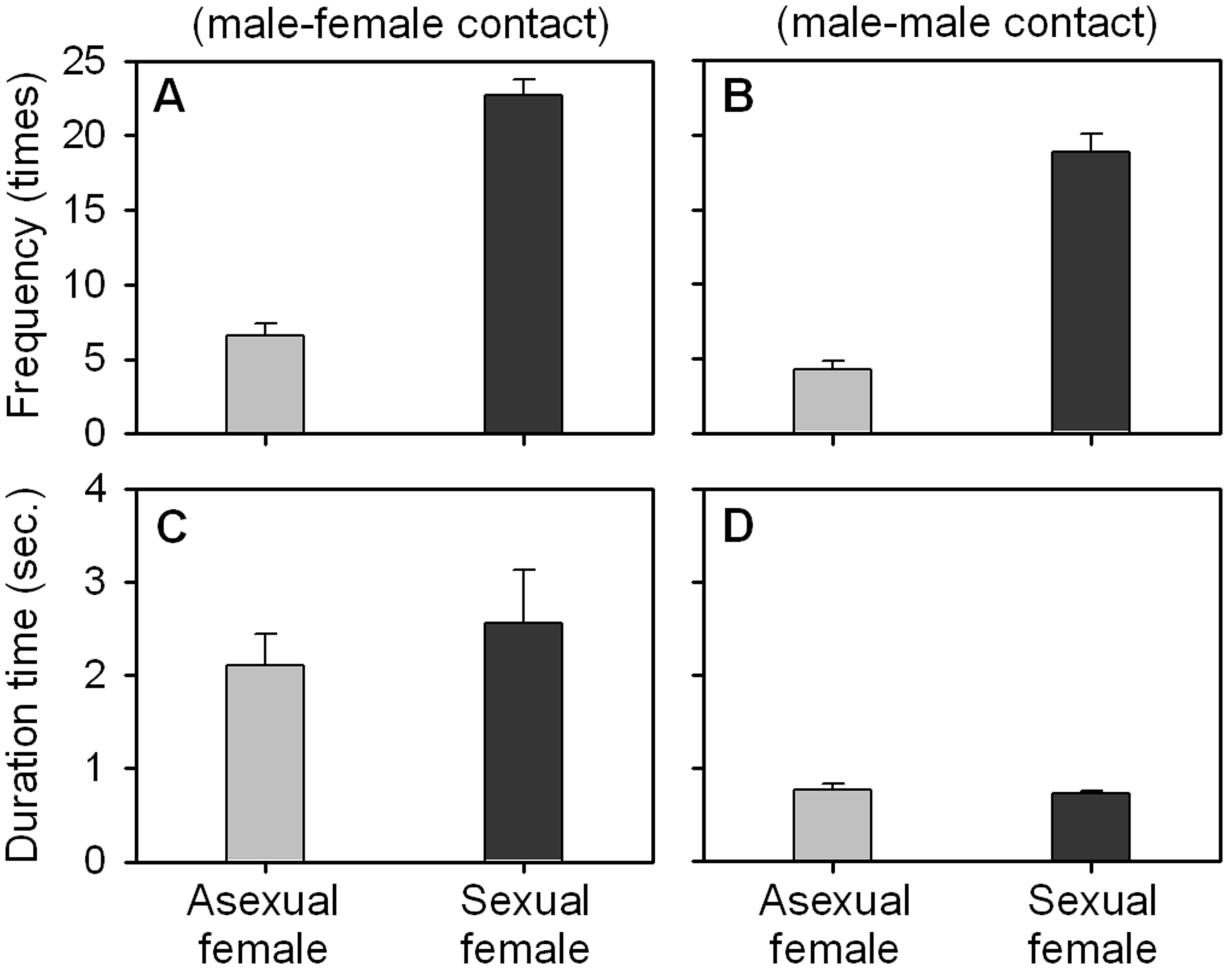
Contact frequencies and duration times of mating and fighting of *Daphnia obtusa* according to reproductive phase of females. Observation was performed for 10 minutes. Each column represents the mean ± SE of 48 replicates.

There was a significant (*p* = 0.000) increase in contact frequency according to the reproductive phase of females. In mating, the mean frequency was low (6.5) in male–asexual female combinations. However, there was a three–fold (22.7) increase when sexual females were put together with males. In addition, males contacted more frequently (*p* = 0.000) with other males when sexual females were present in the same observation chamber (Fig. 5A, B and Table S1). Despite this increase in frequency, the durations of mating and fighting were not dependent upon the sexual conditions of the females (Fig. 5C, D and Table S1).

## Discussion

During mating, the high visibility and unnatural swimming behaviors of males and sexual females make them vulnerable since size, transparency, and pigmentation of prey heavily affect predation rate [44], [45]. The effects of predation on mating behavior have been tested for crustaceans such as decapod *Rhynchocinetes typus*, amphipod *Gammarus duebeni*, and copepod *Cyclops vicinus*. Male *R. typus* under predation risk from fish (*Auchenionchus microcirrhis*) did not change their mating behavior [46], whereas male and female *G. duebeni* show reduced activity leading to less pair formation in the presence of sticklebacks (*Gasterosteus aculaetus*) [47]. *C. vicinus* exposed to *Chaoborus* kairomones show no decrease in mating, although both visual (roach; *Rutilus rutilus*) and tactile (phantom midge; *Chaoborus flavicans*) predators actually consumed more copepods during copulation [48]. Thus, male–asexual female and male–sexual female contacts are undoubtedly risky in nature. Furthermore, fighting between males leads to predation risk.

In the presence of predation risk, *Daphnia* are subjected to a trade–off between mating and producing resting eggs for the next generation or avoiding mating in order to reduce mortality. When *Daphnia* perceives fish kairomones, they swim slowly to reduce the predatory encounter rate [30]. Therefore, kairomones themselves may make encounters between the sexes more difficult. However, our results indicate an increase in mating frequency along with a decrease in fighting in the presence of fish kairomones. This suggests that *Daphnia* obviously take cues from predators and actively change their mating and fighting strategies according to the circumstances. Fast swimming during mating and fighting in *Daphnia* appear to be an escape behavior similar to the response against physical turbulence [49]. Sexual reproduction is performed mainly during early summer, a time when predators are ubiquitous and this seasonal variation is hard to reverse until winter. Moreover, the switch to sexual reproduction occurs in response to population levels, and abundant sexual females exist even when there is a high predation risk. In this situation, investment in mating may be the best choice even under the worst conditions since the ultimate goal of both sexes is mating to ensure the transfer of genes.

It is well known that *Daphnia* recognize food concentrations and alter their behavior accordingly [33], [50]. Moreover, recent molecular work has revealed that *Daphnia* have genes encoding gustatory receptors [51]. Under low food conditions, *Daphnia* swim slowly possibly to save energetic costs [32], and as a result, low food can cause a low encounter rate. In this situation, males tend to avoid unnecessary fighting while mating is not affected. An established low food concentration or short periods of food stress in our experiment did not seem to affect mating, since *Daphnia* often experience low food conditions in the field [52].

In the experiment on the impact of reproductive phase of females, frequencies of male-sexual female contact were higher than those of male-asexual female and male-male contacts. Distinction of the receptiveness of females by males before actual contact is the most important factor in explaining this differential behavior. One feasible way for males to identify sexual females is chemical reception. Male copepods, such as *Euryptemora affinis*, *E. herdmani*, and *Pseudodiaptomus coronatus* are known to detected females or other males without actually making contact using pheromones operated within the genus level [53]. Evidence for chemical interactions between males and females has also been found in the small cladoceran *Chydorus sphaericus*; males swim faster with complex tracks when females are present in the water [54]. However, the use of water–soluble pheromones to assess the reproductive phase of females was not observed in the closely–related species *D. pulicaria* [55]. Similarly, direct observation of the behavior of male *D. magna* to receptive sexual females did not support the presence of sex–related soluble chemicals [56]. If *D. obtusa* indeed does not use chemicals for the distinction of sex and receptive female, other methods such as electrical or mechanical reception displayed by tactile predators can be used for mate selection [57]. *Daphnia* make electrical noises when they swim [58]. In addition, *D. pulicaria* and *D. pulex* produce detectable micro–turbulences larger than their body length when they swim [59], and these are affected by body size [60].

**Figure 6:**
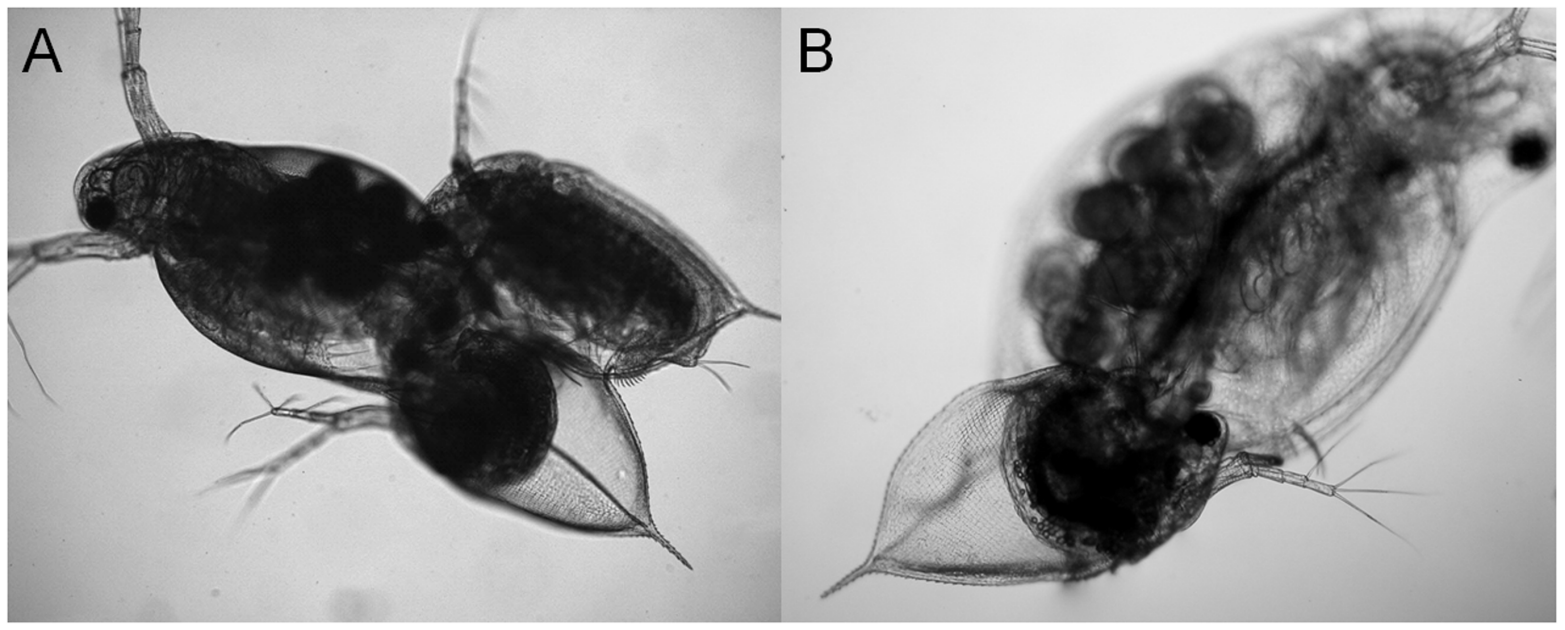
Insemination behaviors of the *Daphnia obtusa* male during contact with asexual female of *D. obtusa* (A) or *D. galeata* (B).

After contact, *D. obtusa* displayed significantly shorter and more consistent fighting duration compared to during mating regardless of conditions (Table S2). It is unclear whether or not such short interactions between males are enough to deter rivals and ensured possibility of mating. Previous studies on the mating behavior of the cladocerans *D. pulicaria* (average 13.8 sec. for mating and 1.6 sec. for fighting) [23] and *M. brachiata* (16∼25 sec. for mating and 1∼3 sec. for fighting) [14] have also confirmed short fighting durations. Therefore, it is suggested that males certainly distinguish the sexes of mates immediately after contact, and cues such as strength of escape behavior, size difference, and carapace morphology are considered. One of the most interesting aspects of the mating behavior of *Daphnia* is whether or not fruitless insemination occurs during male–asexual female contact. A precise insemination strategy according to the receptiveness of females following mate choice before contact can be predicted. Nevertheless, *D. obtusa* males showed insertion into the postabdomen to inseminate asexual *D. obtusa* females and even *D. galeata* females when pairs were carefully transferred and placed under the microscope for observation (Fig. 6). These observations suggest that the species and receptiveness of the female are not critical to copulation itself. This imprecision may be the cause of interspecific hybridization of cladocerans such as *Bosmina* [61], *Daphnia* [62] and *Simocephalus* [63].

Mating and fighting behaviors of *D. obtusa* can be summarized in three steps. 1. Pre–contact: the step of balancing. Factors such as predation risk, food quantity, as well as reproductive phase of females influence the chasing behavior of males. Males determine whether to chase or pass according to the magnitude of swimming turbulence. This enables the primary screening of other small males since adult females are normally larger than the male. 2. Contact: the step of sexing. The male distinguishes the sex of the contacted individual possibly based on differences in size and carapace shape. If the contacted individual is of the same sex, the male detaches immediately. 3. Post–contact: the step of actual copulation. In this step, external factors do not influence copulation and actual copulation is performed regardless of the species and receptiveness of the female. Mating in *Daphnia* does not occur randomly, but rather they assess multiple conditions before contact to maximize mating success. The results were expressed quantitatively (frequency) rather than qualitatively (duration), although the actual increase in the number of resting eggs according to enhanced mating requires further investigation.

## Supporting Information

Table S1 **Results of three–way ANOVA for the impact of three factors and their reciprocal interaction on the mating and fighting behavior of** ***Daphnia obtusa*** **.**
(DOCX)

Table S2 **Results of one–way ANOVA for differences between mating and fighting according to the treatments.**
(DOCX)
