# Supplementary material for: Mating Behavior of Daphnia: Impacts of Predation Risk, Food Quantity, and Reproductive Phase of Females
Source: PLoS One. 2014 Aug 11;9(8):e104545. doi: 10.1371/journal.pone.0104545 (PMC4128813; doi:10.1371/journal.pone.0104545)
Supplement: Table S1 — Results of three–way ANOVA for the impact of three factors and their reciprocal interaction on the mating and fighting behavior of Daphnia obtusa . (DOCX) [file pone.0104545.s001.docx]

**Table S1. Results of three–way ANOVA for the impact of three factors and their reciprocal interaction on the mating and fighting behavior of *Daphnia obtusa.***

| Contact types | Factors | Measurements | | | | | |
| --- | --- | --- | --- | --- | --- | --- | --- |
|  |  | Frequency | | | Duration time | | |
|  |  | d.f. | F | *P* | d.f. | F | *P* |
| Mating | Fish kairomones (K) | 1 | 8.89 | **0.004** | 1 | 1.46 | 0.231 |
|  | Food quantity (F) | 1 | 0.08 | 0.777 | 1 | 1.88 | 0.174 |
|  | Reproductive phase of females (P) | 1 | 190 | **0.000** | 1 | 0.47 | 0.493 |
|  | K*F | 1 | 0.50 | 0.480 | 1 | 1.67 | 0.200 |
|  | K*P | 1 | 0.25 | 0.621 | 1 | 0.00 | 0.964 |
|  | F*P | 1 | 0.13 | 0.724 | 1 | 0.32 | 0.574 |
|  | K*F*P | 1 | 31.4 | **0.000** | 1 | 0.36 | 0.548 |
| Fighting | Fish kairomones (K) | 1 | 16.1 | **0.000** | 1 | 0.10 | 0.757 |
|  | Food quantity (F) | 1 | 13.9 | **0.000** | 1 | 3.15 | 0.079 |
|  | Reproductive phase of females (P) | 1 | 191 | **0.000** | 1 | 0.16 | 0.687 |
|  | K*F | 1 | 0.28 | 0.595 | 1 | 0.55 | 0.460 |
|  | K*P | 1 | 2.44 | 0.122 | 1 | 0.43 | 0.516 |
|  | F*P | 1 | 0.43 | 0.516 | 1 | 1.35 | 0.249 |
|  | K*F*P | 1 | 23.8 | **0.000** | 1 | 0.74 | 0.392 |
