# Supplementary material for: Mating Behavior of Daphnia: Impacts of Predation Risk, Food Quantity, and Reproductive Phase of Females
Source: PLoS One. 2014 Aug 11;9(8):e104545. doi: 10.1371/journal.pone.0104545 (PMC4128813; doi:10.1371/journal.pone.0104545)
Supplement: Table S2 — Results of one–way ANOVA for differences between mating and fighting according to the treatments. (DOCX) [file pone.0104545.s002.docx]

**Table S2. Results of one–way ANOVA for differences between mating and fighting according to the treatments.**

| Conditions | Measurements | Mating *vs*. fighting | | |
| --- | --- | --- | --- | --- |
|  |  | d.f. | F | *P* |
| No fish | Frequency | 1 | 0.16 | 0.692 |
|  | Duration time | 1 | 40.4 | **0.000** |
| Fish | Frequency | 1 | 11.6 | **0.001** |
|  | Duration time | 1 | 9.66 | 0.002 |
| Low food | Frequency | 1 | 6.33 | **0.014** |
|  | Duration time | 1 | 25.1 | **0.000** |
| High food | Frequency | 1 | 0.20 | 0.657 |
|  | Duration time | 1 | 11.5 | **0.001** |
| Asexual female | Frequency | 1 | 4.70 | **0.033** |
|  | Duration time | 1 | 14.7 | **0.000** |
| Sexual female | Frequency | 1 | 5.86 | **0.017** |
|  | Duration time | 1 | 10.4 | **0.002** |
